# Demographic factors, attitude and knowledge of persons with special needs towards COVID-19 in Nigeria: Implications for counselling and social policy

**DOI:** 10.35241/emeraldopenres.13800.2

**Published:** 2020-09-30

**Authors:** Kelechi Uchemadu Lazarus, David Adebayo Oluwole

**Affiliations:** 1 Department of Special Education, Faculty of Education, University of Ibadan, Ibadan, Oyo State, Nigeria; 2 Department of Guidance and Counselling, Faculty of Education, University of Ibadan, Ibadan, Oyo State, Nigeria

**Keywords:** Demographic factors, Attitude, Knowledge, Persons with special needs, COVID-19, Counselling and social policy

## Abstract

This study investigated demographic factors, attitude and knowledge of persons with special needs towards COVID-19 in Nigeria between 12 ^th^ and 25 ^th^ May, 2020. This cross-sectional online survey was conducted among 72 persons with special needs purposively selected from the six geo-political zones in Nigeria. A questionnaire comprising questions on demographic information (three), knowledge (24) and attitude (28) towards COVID-19 was completed via Google forms by the participants (r = 0.78). There were more males (51, 70.8%) than females (21, 29.2%) and the most common age group was 34-44 years (37.5%). The number of participants with a hearing impairment was 34 (47.2%) and the number with a visual impairment was 26 (36.1%). The results indicate adequate knowledge about the characteristics of COVID-19. It was found that 98.6% of the participants had heard about COVID-19; 94.4% were aware that COVID-19 is a contagious disease, 91.7% stated that COVID-19 is a virus and 88.9% reported correctly that the incubation period is 3-14 days. The knowledge about symptoms of COVID-19 among participants was high (x = 2.63; participants obtained 87.8% of the total achievable score for these questions). The knowledge about prevention and control of COVID-19 among participants needs was very high (x = 2.77; participants obtained 92.3% of the total achievable score for these questions). Attitude of participants towards the COVID-19 outbreak was positive and above average (x = 2.84). However, participants reported that it is hard to get palliatives or financial support from others during COVID-19 lockdown (52.8%) and that they feel frustrated by the uncaring attitude of the government towards them during COVID-19 lockdown (55.6%). On this basis, counselling and social policy implications were suggested including the provision of palliatives by the government and the need for widespread enlightenment among individuals with special needs on prevention of COVID-19.

## Introduction

The novel coronavirus disease discovered in 2019 (COVID-19) belongs to the Coroviridae family and is an RNA virus ( Ochei & Kolhatkar, 2017). COVID-19 is a new strain of coronavirus that has not been previously identified in humans. It was first discovered in Wuhan city, China in December 2019. It is an infectious disease with symptoms ranging from fever, headache, sore throat, tiredness, dry cough, chest pain, to shortness of breath and breathing difficulties. This disease can cause pneumonia, severe acute respiratory syndrome, kidney failure and even death. The World Health Organization (WHO) announced a name for the new coronavirus disease, COVID-19, on 1 ^st^ February 2020 and declared COVID-19 as a pandemic on 12th March 2020 as a result of the fast rate at which it was spreading to countries of the world ( WHO Regional Office for Europe, March 12 2020). As of Wednesday, 3 ^rd^ June 2020, statistics on COVID-19 showed that there are now 6,474,559 confirmed coronavirus cases globally with 3,083,688 (89%) recoveries and discharged cases and 382,921 (11%) fatalities ( Worldometer, June 3 2020). 

The Federal Ministry of Health in Nigeria reported that Nigeria recorded its index case on 27 ^th^ February 2020 through an Italian expatriate who works in Nigeria and returned from Milan, Italy, to Lagos, Nigeria, on the 25 ^th^ February 2020 ( Nigeria Centre for Disease Control, February 28 2020). Since the index case of COVID-19 in Nigeria, the cases have been on increase. As of June 1 ^st^ 2020, updated reports of the COVID-19 outbreak by the Nigeria Centre for Disease Control (NCDC) showed that 65,885 persons have been tested, and there are 10,578 confirmed cases spread across 36 states of the federation including the Federal Capital Territory with 3,122 recoveries/discharged cases and 299 (~3%) confirmed fatalities. The most affected group are those between 31–40 years (24%), and there are more cases in males than females, with 7,133 (67%) cases in males and 3,445 (33%) cases in females ( Nigeria Centre for Disease Control, June 1 2020).

Older people and people with underlying medical conditions such as those with special needs are the most vulnerable to COVID-19 infection; however, people of all ages are likely to be infected by COVID-19. The WHO indicated that standard recommendations to prevent infection spread include regular hand washing, covering of the mouth and nose when coughing and sneezing as well as thoroughly cooking meat and eggs. Individuals are also advised to avoid close contact with anyone showing symptoms of respiratory illness such as coughing. There is currently no specific treatment or vaccine for COVID-19 but most people infected with COVID-19 recover over time; for mild cases this takes approximately two weeks, but for severe and critical cases it can take three to six weeks for patients to show signs of recovery ( WHO Regional Office for the Eastern Mediterranean, 2020).

Persons with special needs experience mild to severe degrees of impairments, which pose difficulties for the way they perform certain tasks in society. Such persons include those with a learning disability, speech or language impairment, intellectual disability, emotional disturbance, autism, hearing impairment, visual impairment, deaf-blindness, orthopaedic impairment, traumatic brain injury, other health impairment, multiple disabilities and developmental delay ( Smith, 2007). These individuals constitute about 15% of the world’s population (a billion people) and within this group, an estimate of between 110 million and 190 million people experience significant disabilities ( World Bank, April 1 2020). With the outbreak of the coronavirus disease in the world, the way several activities are performed among individuals without special needs is rapidly changing. Thus, the delivery of services to specific categories of persons with special needs requires urgent attention and care. The present study elicited responses from different categories of persons with special needs (hearing impairment, visual impairment, physical disabilities, deaf-blindness, emotional disorder, speech and language disorder, multiple disabilities). This is because with the emergence of COVID-19 and its attendant challenges, there is the need to support persons with special needs to effectively cope with the new normal.

In March 2020, the WHO released their latest statistics on hearing loss, stating that over 5% of the world’s population – or 466 million people, including 34 million children – are currently living with a hearing impairment globally, with a projection that the estimate will increase to over 900 million people in 2050 – or one in every ten people. Buttressing this data, Ademokoya (2020) reiterated that 80% of global hearing loss burden is in low- and middle-income countries including Nigeria. Ademokoya explained that hearing loss among Nigerians have increased from 7.3 million in 1999 to an estimate of 8.5 million in 2020. Reasons put forward for this alarming number include poverty, lack of awareness, exposure to gunshots through wars and violence, noise pollution, abuse of ear pieces, drug abuse, genetic factors, psychological factors, poor lifestyle choices, illnesses, injuries, misuse of sharp objects, accidents, and diet. Hearing impairment impedes a person’s ability to receive auditory signals. Mild, moderate or severe hearing loss alters the manner in which a person learns, communicates and integrates in society. Hearing impairment can result in social isolation and stigmatization as well as difficulties in obtaining, performing and keeping an occupation. These characteristics therefore make the delivery of major services in terms of education, social, health and sport somewhat different for persons with hearing impairment.

The estimate for the incidence of visual impairment is placed at 2.2 billion people globally by the WHO. It is stated that at least 1 billion people within this group have a vision impairment that could have been prevented or has yet to be addressed. Persons with visual impairment experience difficulty in the ability to receive information visually. The adverse effects of partial to total sightedness and low vision include difficulties with physical mobility, motor skills, emotional and psychological maladjustments and a restructuring of the range of life experiences ( Abang, 1992). To access most services in society there is a need to provide some types of adaptations and adjustments. Without these specialized services, persons with special needs suffer a lot of disadvantages. Depending on the extent of the disability, persons with hearing impairments communicate with others through the use of sign language, speech reading or other specialized strategies. Partially sighted individuals would require adaptive devices such as magnifying glasses to access learning materials whilst those who are blind do not use vision to learn; instead they make use of braille, computers adapted for their use, and other assistive devices.

Other categories of persons with special needs considered in this study are hereby described. Persons with physical disabilities include those with impairments caused by a congenital anomaly, impairments caused by disease such as poliomyelitis, bone tuberculosis, and impairments from other causes (such as cerebral palsy, amputations and fractures or burns that cause contractures) ( Key Terms to Know in Special Education Oct 8, 2017). Deaf-blindness is a dual disability that involves both hearing and vision problems. Although most persons in this category have some residual hearing and/or vision their world can be exceptionally restricted ( Smith, 2007). Persons with emotional disabilities primarily exhibit behaviour problems which significantly fall beyond their cultural and age norms on externalizing dimension (for instance, aggression) and internalizing dimension (for example, anxiety) ( Heward, 2013). Speech and language disorders include problems in communication such as stuttering, articulation difficulties, language impairment, or a voice impairment which affects an individual’s educational and social functioning. According to Smith (2007), persons with multiple disabilities have more than one condition that influences learning, independence, and the range of intensive and pervasive supports the individual and the family require. Whereas multiple disabilities include intellectual disability-blindness or intellectual disability-orthopaedic impairment it does not include deaf-blindness.

The need to address the unique difficulties such as in the area of hearing and vision, cognitive functioning, mobility, and learning as well as attitudinal barriers to health care for persons with special needs ( United Nations Flagship Report on Disability and Development 2018) is germane to the education and the general welfare of these persons. Hence, the United Nations Human Rights Office of the High Commissioner issued a joint statement through the Chair of the United Nations Committee on the Rights of Persons with Disabilities where it postulated a ten-point policy in favour of persons with special needs during the COVID-19 outbreak. Among those statements is the fact that measures should be put in place to ensure that the lives and rights of persons with disabilities are appropriately protected in the face of the COVID-19 pandemic. States were also called upon to ensure the inclusion and effective participation of persons with disabilities. States were advised to prevent discriminatory denial of health care or life-saving services, food or fluids on the basis of disability.

The extent to which member nations of the United Nations are working towards reducing the impact of COVID-19 on persons with disabilities is yet to be fully grasped. In May 2020 stakeholders in Nigeria, including educators and persons with special needs themselves showed disapproval towards the government concerning its unimpressive practice towards persons with COVID-19 within the education sector. The Punch News (May 25 2020) reported that the Federal Government of Nigeria did not make “special provisions” for electronic learning for persons with special needs while all schools remain closed due to the COVID-19 pandemic. This is a clear indication of negligence on the part of the government and promotion of the culture of excluding those already excluded. More so, according to the Office for National Statistics (20 August 2020) persons with disabilities in Great Britain reported being more worried than persons without disabilities about the effect of COVID-19 on their well-being, health, access to healthcare for non-coronavirus related issues and access to groceries, medication and essentials. Going by these reports, it could be inferred that there is an imperative need for counselling and social policy implications for persons with special needs during the COVID-19 pandemic.

 Studies such as Wu *et al.* (2009) and Hussain *et al.* (2012) indicated that there is a connection between an individuals’ level of knowledge about an infectious disease and the practices he or she adopts in response to disease prevention and management. Zhong *et al.* (2020) found that the COVID-19 knowledge score of participants was significantly associated with a lower likelihood of negative attitudes and preventive practices towards COVID-19. A relatively high socio-economic status, especially among females, was linked to possessing better COVID-19 knowledge, favourable attitudes and appropriate practices towards COVID-19. Among Iranians, Erfani *et al.* (2020) found that knowledge, attitude and practice towards the COVID-19 outbreak were high, positive and appropriate, respectively. A notable correlation was found between females within the higher age bracket and those with a higher education level and knowledge, attitude and practice toward COVID-19. In the same vein, males, single people, persons in professions not related to health care and those with lower levels of education had lower COVID-19 knowledge scores.

During the first week of the COVID-19 lockdown by the Nigerian government from March 28 to April 4 2020, Olapegba *et al.* (2020) conducted a preliminary investigation on COVID-19 and found that Nigerian residents possessed high knowledge of the disease, which was typically obtained from traditional media. Most respondents perceived that COVID-19 is a deadly disease and that with regular hand washing and social distancing the spread of COVID-19 could be contained. It was recommended that an intensive result-oriented campaign would go a long way in removing misconceptions held by Nigerian residents and promote precautionary measures.

 Studies such as Tao (2003) and Person *et al.* (2004) reported on the significance of understanding the impact of knowledge and attitudes towards infectious diseases like severe acute respiratory syndrome (SARS). It was found that both knowledge and attitudes had an influence on the way individuals show fear and other emotions towards infectious diseases and also hinders efforts that can be made to contain the spread of the virus. Most studies ( Erfani *et al.* 2020; Olapegba *et al.*, 2020; Zhong *et al.*, 2020) on attitude, knowledge and health seeking behaviours towards COVID-19 have addressed the needs of persons without special needs. Therefore, limited knowledge is available among persons with special needs, a gap this preliminary investigation in Nigeria hopes to fill. This study set out to investigate the demographic characteristics and level of attitude and knowledge towards persons with special needs in Nigeria during the period of the COVID-19 outbreak and lockdown in Nigeria.

## Research questions

1. What are the demographic characteristics of the respondents?
2. What is the level of knowledge about the general characteristics of COVID-19 among persons with special needs?
3. What is the level of knowledge about the symptoms of COVID-19 among persons with special needs?
4. What is the level of knowledge about prevention and control of COVID-19 among persons with special needs?
5. What is the attitude of persons with special needs towards the COVID-19 outbreak and lockdown?

## Methods

### Ethical statement

The researchers obtained ethical approval from the Faculty of Education Ethical Board, University of Ibadan as well as written informed consent from participants before the study was conducted. Seventy-two (72) persons with special needs consented to provide the required information. None of the participants have disabilities that could affect their abilities to provide informed consent.

### Design

This study adopted a cross-sectional survey research design. Data was collected between 12 ^th^ and 25 ^th^ May 2020 using an online structured questionnaire.

### Participants

The study’s inclusion criteria were as follows:

- Participants are persons living with special needs
- Participants are persons who can read and write simple English
- Participants are willing and ready to participate in the study without coercion
- Participants are versatile with internet surveys

For populations that are large as in the case of this study, the formula developed by Cochran (1963), as cited in Israel (2003) to yield a representative sample for proportions was used.

The formula is depicted below:

$$n_{0}=\frac{z^{2}pq}{e^{2}}$$

where, *n _0_* is the sample size, *z* is the selected critical value of desired confidence level (95%, it is 1.96), *p* is the estimated proportion of an attribute of disability that is present in the Nigerian population, which is 27 million according to the most recent national prevalence ( Umeh & Adeola, 2016). In this present study, about 50% of persons with special needs who can read and write represent 0.054% of the entire population (in this study, the estimated proportion is translated into 0.054%), *q* = 1− *p* and *e* is the desired level of precision (±5% precision, that is 0.05).

Accordingly, 

$$\text{n}=\frac{1.96^{2}\times 0.010\times \left(1-0.010\right)}{0.52^{2}}=\frac{0.1983}{0.0025}$$

n= 79.32 which was approximated to 79

Using a Google online survey, a total of 72 respondents was garnered which is representative of the population of individuals with special needs who could read and write. Using a purposive sampling technique, a total of 72 persons with special needs (hearing impairment, visual impairment, physical disabilities, deaf-blindness, emotional disorder, speech and language disorder and multiple disabilities) were sampled from the six geopolitical zones in Nigeria (precisely, from 17 states out of 36 states including the Federal Capital Territory, Abuja).

Before recruiting participants, the researchers acquired the contact details of persons with special needs in terms of names, phone numbers and email addresses. Lecturers, teachers of persons with special needs, sign language interpreters, parents of special need persons, public relation officers of the association of deaf persons, friends and school mates, fellow church members and co-workers of participants in various towns and cities across the country were contacted to assist in recruitment through direct phone calls, emails, texts, and WhatsApp messages. Only persons with special needs who consented in writing to participate in the study were recruited, hence participation was purely voluntary.

### Procedure

Since it was not possible to conduct a community-based nation-wide research survey in this time of COVID-19 lockdown, the researchers chose to collect the data through the Google online survey platform. Access to the web-page was shared through various social media platforms such as WhatsApp and Facebook to the disabled individuals who served as the participants. Through the link, the participants could view the questions simply by clicking on it and could then answer the questions. Although the questionnaire could be filled out within 10 to 13 minutes by individuals without special needs during a pilot test, participants with special needs reported that the questionnaire took them about 35 to 45 minutes to complete.

### Study instruments

This study used the “Questionnaire on Demographic Factors, Attitude, and Knowledge of Persons with Special Needs towards COVID-19” (QDFAKPSNC). The researchers made reference to existing instruments utilized in a related study ( Erfani *et al.,* 2020). These researchers designed their questionnaires based on WHO training material for detection, prevention, response and control of COVID-19. Considering the special needs of the participants, the researchers made a few adaptations to the instruments, especially in the section on attitude. For instance, whereas a question like “I believe COVID-19 is a serious disease” was taken from the attitude domain of the Erfani *et al.* (2020) questionnaire; questions such as “I feel worried that I do not have assistive technology e.g. hearing aids, mobility canes, internet data, etc. to access information on COVID-19” and “My disability is a constant source of frustration to me during the COVID-19 period” were newly included in the attitude domain of the present study questionnaire. Thus, there are 28 items in the attitude domain as opposed to 15 items in the attitude domain in the Erfani *et al.* (2020) questionnaire. The QDFAKPSNC has different segments covering demographic information of participants (three questions), knowledge (24 questions) and attitude (28 questions) towards COVID-19 accordingly.

Out of the 24 questions that were used to measure knowledge, six questions elicited responses on the general characteristics of COVID-19, including the cause of the disease, the incubation period and how it is treated. A further six questions centred on knowledge about the symptoms of the disease, while twelve questions were on the prevention and control of the disease. These questions were answered on a true/false basis with an additional “No opinion” option. A “true” answer was assigned three points, “false” was assigned one point and “No opinion” was assigned two points. Based on this, the total score achievable for knowledge about the symptoms of COVID-19 ranges from six to 18 (see Table 2). There were 12 questions on knowledge about prevention and control of COVID-19. A score of 55–100% denotes high knowledge, 40–54% denotes low knowledge and below 40% denotes low knowledge of COVID-19.

The total score achievable in this section ranges from 12 to 36. Subsequently, a high score denotes a better knowledge of COVID-19. There were 28 questions used to elicit responses about the attitude of persons with special needs towards COVID-19. The scoring was similar to that of the knowledge section with one, two, three, and four points assigned to “Strongly disagree”, “Disagree”, “Agree” and “Strongly Agree”, respectively. Likewise, a high score signifies a positive attitude and vice versa. A score value of 2.5–4.0 signifies positive attitude of persons with special needs towards COVID-19, while a score value below 2.5 signifies low attitude.

 In a pilot testing effort, the questionnaires were administered to six individuals from the Department of Special Education, University of Ibadan. The data were analysed using SPSS 24.0. The Cronbach alpha statistics yielded a reliability coefficient index of 0.78. This shows the scale is reliable.

### Data analysis

Descriptive statistics of frequency counts, percentages, means and standard deviations were computed for participants’ demographic factors and to derive participants’ knowledge and attitude towards COVID-19. Data analyses were conducted with SPSS version 20 ( IBM Corp, 2011).

## Results

A total of 72 questionnaires that were completely filled were analysed and the results are as follows:

### Research question 1: What are the demographic characteristics of the respondents?

The demographic description of the participants in Table 1 shows that male participants are in the majority, with 51 males (70.8%) and 21 females (29.2%). More than half of the participants were aged 34–44 years (37.5%). There were 34 participants with a hearing impairment (47.2%) and 26 with a visual impairment (36.1%). Other special needs were physical disabilities (6.9%) deaf-blindness (2.8%), emotional disorder (4.2%), speech and language disorder (1.4%) and multiple disabilities (1.4%) ( Lazarus & Oluwole, 2020).

**Table 1.** Demographic characteristics of participants.

Variables	Categories	Frequency (n = 72)	Percent (%)
Gender	Male Female	51 21	70.8 29.2
Age	15 − 24 25 − 34 34 − 44 45 and above	11 21 27 13	15.3 29.2 37.5 18.0
Disability type	Hearing impairment Visual impairment Physical disabilities Deaf-blindness Emotional disorder Speech & lang. disorder Multiple disabilities	34 26 5 2 3 1 1	47.2 36.1 6.9 2.8 4.2 1.4 1.4

**Table 2.** Knowledge about the general characteristics of COVID-19 among persons with special needs.

S/N	Items	Responses	
		Frequency	Percent (%)
1	I have heard about COVID-19? a. True b. False c. No opinion	71 1 0	98.6 1.4 0
2	COVID-19 is a contagious disease. a. True b. False c. No opinion	68 1 3	94.4 1.4 4.2
3	Which of the following is the cause of COVID-19? a. Bacteria b. Virus c. Fungi d. Parasite e. Immunodeficiency f. No opinion	1 66 1 0 2 2	1.4 91.7 1.4 0 2.8 2.8
4	How long is the incubation period of the disease? a. Less than 2 days b. 2 to 5 days c. 3 to 14 days d. No opinion	0 6 64 2	0 8.3 88.9 2.8
5	Which of the following is the treatment for COVID-19? a. Symptomatic therapy b. Antibiotics c. No treatment d. No opinion	25 10 19 18	34.7 13.9 26.4 25
6	In which age group is the disease more dangerous? a. Under 15 years b. 15 to 30 years c. 30 to 50 years d. Above 50 years e No opinion	1 1 8 54 8	1.4 1.4 11.1 75 11.1

### Research question 2: What is the level of knowledge about the general characteristics of COVID-19 among persons with special needs?

With respect to knowledge about COVID-19 general characteristics among persons with special needs, it was revealed in Table 2 that 98.6% of them have heard about COVID-19; 94.4% are aware that COVID-19 is a contagious disease, 91.7% stated that COVID-19 is a virus and 88.9% reported correctly that the incubation period is 3–14 days. There was a wide variation of responses on what the treatment measures for COVID-19, as 34.7% of the participants stated that symptomatic therapy is the treatment for COVID-19, 13.9% stated that antibiotics are the treatment, while 26.4% and 25% stated that there is no treatment or had no opinion on treatment for COVID-19, respectively. Regarding the age group in which the disease is most dangerous, 75% responded that it is among those “above 50 years” These results indicate adequate knowledge about the general characteristics of COVID-19 among persons with special needs in Nigeria.

### Research question 3: What is the level of knowledge about symptoms of COVID-19 among persons with special needs?

On knowledge about the symptoms of COVID-19 among persons with special needs, it was revealed in Table 3 that the majority of the respondents agreed that cough is a symptom of COVID-19 (93.1%), followed by sore throat (88.9%) and fever (84.7%). However, 48.6% stated that diarrhoea or constipation is a symptom of COVID-19. Also, when infection with COVID-19 is suspected, fever will be measured primarily (88.9%). These statements are correct according to WHO. Overall, knowledge about symptoms of COVID-19 among persons with special needs is high, with a weighted mean score of 2.63 out of 3.00. In addition, based on the total achievable score in this section, which ranges from 6–18, the mean score for knowledge about symptoms of COVID-19 obtained by participants was 15.78, which correspond to 87.8% of the total achievable score. This implies that knowledge about the symptoms of COVID-19 is high. A majority (78.5%) had fairly high knowledge about the symptoms of COVID-19, while the remaining participants (21.5%) had low knowledge about symptoms of COVID-19.

**Table 3.** Knowledge about symptoms of COVID-19 among persons with special needs.

S/N	Items	True, n (%)	False, n (%)	No opinion, n (%)	x̅	S.D.
7	Cough is a symptom of COVID-19	67 (93.1%)	4 (5.6%)	1 (1.4%)	2.88	0.47
8	Sore throat is a symptom of COVID-19	64 (88.9%)	6 (8.3%)	2 (2.8%)	2.81	0.57
9	Fever is a symptom of COVID-19	61 (84.7%)	9 (12.5%)	2 (2.8%)	2.72	0.67
10	Headache is a symptom of COVID-19	58 (80.6%)	10 (13.9%)	4 (5.6%)	2.67	0.71
11	Body pain is a symptom of COVID-19	54 (75%)	12 (16.7%)	6 (8.3%)	2.58	0.76
12	Diarrhoea or constipation is a symptom of COVID-19	35 (48.6%)	28 (38.9%)	9 (12.5%)	2.10	0.93
Weighted mean: 2.63						

### Research question 4: What is the level of knowledge about prevention and control of COVID-19 among persons with special needs?

On knowledge about the prevention and control of COVID-19 among persons with special needs, Table 4 shows that 94.4% stated that the disease can be transmitted directly through coughing, and that the disease can be transmitted directly through contact with infected surfaces. Furthermore, 93.1% agreed that the disease can be transmitted directly through contact with infected persons (handshaking, hugging, kissing), the disease is more dangerous in older individuals, and the disease is more dangerous in people with weakened immune systems. The vast majority (91.7%) stated that the prevalence of COVID-19 disease is increasing in Nigeria. Knowledge about the prevention and control of COVID-19 among persons with special needs is very high (x = 2.77). In addition, based on the total achievable score in this section, which ranges from 12 to 36, the mean score for knowledge about prevention and control of COVID-19 obtained by participants was 33.24, which corresponds to 92.3% of the total achievable score. This implies that the knowledge about prevention and control of COVID-19 was very high. A majority (85%) had high knowledge about the prevention and control of COVID-19, while the remaining participants (15%) had moderate or low knowledge about the prevention and control of COVID-19.

**Table 4.** Knowledge about prevention and control of COVID 19 among persons with special needs.

S/N	Items	True, n (%)	False, n (%)	No opinion, n (%)	x̅	S.D.
13	The disease can be transmitted directly through cough	68 (94.4%)	1 (1.4%)	3 (4.2%)	2.93	0.30
14	The disease can be transmitted directly through contact with infected surfaces	68 (94.4%)	2 (2.8%)	2 (2.8%)	2.92	0.36
15	The disease can be transmitted directly through contact with infected persons (handshaking, hugging, kissing)	67 (93.1%)	3 (4.2%)	2 (2.8%)	2.90	0.43
16	The disease is more dangerous in old individuals	67 (93.1%)	3 (4.2%)	2 (2.8%)	2.90	0.43
17	The disease is more dangerous in people with cancer, diabetes, and chronic respiratory diseases	66 (91.7%)	2 (2.8%)	4 (5.6%)	2.90	0.39
18	In suspecting infection with COVID-19, primarily I will measure fever	64 (88.9%)	1 (1.4%)	7 (9.7%)	2.88	0.37
19	The prevalence of COVID-19 disease is increasing in Nigeria	66 (91.7%)	3 (4.2%)	3 (4.2%)	2.88	0.44
20	The disease is more dangerous in people with weakened immune systems	67 (93.1%)	4 (5.6%)	1 (1.4%)	2.88	0.47
21	Washing hands with water and soap can eliminate the disease cause	62 (86.1%)	7 (9.7%)	3 (4.2%)	2.76	0.61
22	In suspecting infection with COVID-19, I will avoid unnecessary daily activities	61 (84.7%)	7 (9.7%)	4 (5.6%)	2.75	0.62
23	The disease is more dangerous in pregnant women	40 (55.6%)	13 (18.1%)	19 (26.4%)	2.38	0.77
24	The disease can be transmitted directly through the consumption of contaminated dairy and meat	38 (52.8%)	24 (33.3%)	10 (13.9%)	2.19	0.91
**Weighed mean = 2.77**						

### Research question 5: What is the attitude of persons with special needs towards the COVID-19 outbreak and lockdown?

Table 5 shows that with respect to the attitude of persons with special needs towards the COVID-19 outbreak, the majority of the respondents strongly agreed that COVID-19 is a serious disease (66.7%), COVID-19 can be avoided by proper preventive measures (54.2%), authorities should quarantine COVID-19 patients in special hospitals (50.0%), and health education can help prevent COVID-19 (51.4%). Further, the participants strongly agreed that they feel authorities should restrict travel to and from COVID-19 areas (50.0%), that it is hard getting palliatives or financial and material support/help from others during COVID-19 lockdown (52.8%) and that they feel frustrated by the uncaring attitudes of the government towards people living with disabilities during COVID-19 lockdown (55.6%). Most of them agreed that many persons with disabilities do have specific underlying conditions that make the disease more dangerous for them (45.8%) and that they are worried that they do not have assistive technology; for example, hearing aids, mobility canes, internet data, etc. to access information on COVID-19 (44.4%). Overall, the attitude of persons with special needs towards the COVID-19 outbreak is positive with the weighted mean as 2.84 against the criterion mean of 2.50.

**Table 5.** Attitude of persons with special needs towards the COVID-19 outbreak.

S/N	Items	SD	D	A	SA	x̅	S.D.
25	I believe COVID-19 is a serious disease	5 6.9%	0 0%	19 26.4%	48 66.7%	3.53	0.82
26	I believe COVID-19 can be avoided by proper preventive measures	4 5.6%	1 1.4%	28 38.9%	39 54.2%	3.42	0.78
27	I feel authorities should quarantine COVID-19 patients in special hospitals	4 5.6%	3 4.2%	29 40.3%	36 50.0%	3.35	0.80
28	I believe health education can help prevent COVID-19	6 8.3%	2 2.8%	27 37.5%	37 51.4%	3.32	0.88
29	I feel authorities should restrict travel to and from COVID-19 areas	5 6.9%	3 4.2%	28 38.9%	36 50.0%	3.32	0.85
30	I feel it is hard getting palliatives/financial and material support/help from others during COVID-19 lockdown	4 5.6%	8 11.1%	22 30.6%	38 52.8%	3.31	0.83
31	I feel frustrated by the uncaring attitudes of the government towards people living with disabilities during COVID-19 lockdown	7 9.7%	6 8.3%	19 26.4%	40 55.6%	3.28	1.01
32	I feel many persons with disabilities have specific underlying conditions that make the disease more dangerous for them	3 4.2%	9 12.5%	33 45.8%	27 37.5%	3.17	0.80
33	I feel that early detection of COVID-19 can improve treatment and outcome	11 15.3%	4 5.5%	20 27.8%	37 51.4%	3.15	1.08
34	I will be willing to serve as a volunteer staff for NCDC if given the opportunity	4 5.6%	14 19.4%	27 37.5%	27 37.5%	3.07	0.89
35	I feel worried that I do not have assistive technology e.g. hearing aids, mobility canes, internet data, etc. to access information on COVID-19	9 12.5%	10 13.9%	21 29.2%	32 44.4%	3.06	1.04
36	It is my opinion that COVID-19 is transmitted to people from wild animals	8 11.1%	6 8.3%	34 47.2%	24 33.3%	3.03	0.93
37	Persons with special needs do not have the emotional help and support we need from society during COVID-19 lockdowns	12 16.7%	7 9.7%	25 34.7%	28 38.9%	2.96	1.07
38	I feel that those who escape from isolation centres should be prosecuted	9 12.5%	15 20.8%	25 34.7%	23 31.9%	2.86	1.00
39	I think my family, friends, caregivers are caring during COVID-19 lockdowns	11 15.3%	6 8.3%	38 52.8%	17 23.6%	2.85	0.95
40	My disability is a constant source of frustration to me during the COVID-19 period	9 12.5%	14 19.4%	29 40.3%	20 27.8%	2.83	0.97
41	I believe that COVID-19 is curable	11 15.3%	7 9.7%	38 52.8%	16 22.2%	2.82	0.95
42	Lack of quality information about COVID-19 is a major barrier to my wellbeing	9 12.5%	22 30.6%	24 33.3%	17 23.6%	2.68	0.97
43	My peers without disability have been fully equipped to protect themselves from COVID-19 compared to persons with special needs	15 20.8%	15 20.8%	23 31.9%	19 26.4%	2.64	1.08
44	I experience a great difficulty with acquiring information about COVID-19	16 22.2%	20 27.8%	24 33.3%	12 16.7%	2.44	1.01
45	I feel nervous that I could contract COVID-19	20 27.8%	13 18.1%	29 40.3%	10 13.9%	2.40	1.04
46	I often hesitate to seek information about COVID-19 disease	17 23.6%	21 29.2%	23 31.9%	11 15.3%	2.39	1.00
47	COVID-19 readiness measures, response plans and their impact in Nigeria is okay	18 25.0%	16 22.2%	31 43.1%	7 9.7%	2.38	0.96
48	I think that the awareness considering COVID-19 in society is sufficient.	19 26.4%	15 20.8%	29 40.3%	9 12.5%	2.34	1.01
49	I believe that COVID-19 disease results in death in all cases	19 26.4%	21 29.2%	21 29.2%	11 15.3%	2.33	1.08
50	My special needs condition often prevents me from acquiring personal hygiene skills	21 29.2%	20 27.8%	19 26.4%	12 16.7%	2.30	1.06
51	I feel angry in my struggle to understand COVID-19	18 25.0%	22 30.6%	22 30.6%	10 13.9%	2.30	1.00
52	It is my opinion that COVID-19 can be treated at home	25 34.7%	25 34.7%	16 22.2%	6 8.3%	2.04	0.95
**Weighted Mean = 2. 84 Criterion Mean = ≥2.50**							

SD, strongly disagree; D, disagree; A, agree; SA, strongly agree; NCDC, Nigeria Centre for Disease Control.

## Discussion

The demographic data in this study have revealed that many participants had hearing and vision problems. Within these two categories, there were more persons with a hearing impairment than those with a visual impairment. This finding is in agreement with the observation made by Ademokoya (2020), who estimated that 8.5 million Nigerians live with various kinds of hearing impairment that are caused by unnecessary exposure of individuals to gunshots/violence/wars, noise pollution, and abuse of ear pieces, drugs and poor lifestyle choices. In other words, if these societally orchestrated experiences are monitored, there is a possibility of reducing the number of persons with a hearing impairment in Nigeria. This has implications for counselling Nigerian youths, parents, educators, religious leaders and the general public. The Nigerian government can draw inferences from this finding for designing social policy.

Our present finding corroborated that of Erfani *et al.* (2020), who found that levels of knowledge, attitude and practice during COVID-19 outbreak were high, positive and appropriate, respectively. It also supported the study of Olapegba *et al.* (2020), who found that Nigerian residents possessed a high level of knowledge of the disease, which was typically obtained from traditional media. However, the findings of this study on knowledge as well as health seeking behaviours of persons with special needs in Nigeria corroborated findings of Wu *et al.* (2009) and Hussain *et al.* (2012), who indicated that there is a connection between an individual’s level of knowledge about an infectious disease and the practices they adopt in response to disease prevention and management. Zhong *et al.* (2020) found that high COVID-19 knowledge score was significantly associated with a lower likelihood of negative attitudes and preventive practices towards COVID-19. This present study sustained the hypothesis that the knowledge of persons with special needs in Nigeria during the period of the COVID-19 outbreak in Nigeria is high, although they feel the government has not been fair to them.

Surprisingly, persons with special needs in Nigeria during the period of the COVID-19 outbreak have an above average positive attitude to COVID-19. This finding reinforced the research outcomes of Tao (2003) and Person *et al.* (2004), who reported on the significance of understanding the impact of knowledge and attitudes towards infectious diseases like SARS. In our present study, the majority of the respondents strongly agreed that COVID-19 is a serious disease (66.7%), that COVID-19 can be avoided by proper preventive measures (54.2%), that authorities should quarantine COVID-19 patients in special hospitals (50.0%), and that health education can help prevent COVID-19 (51.4%). Further, the participants strongly agreed that they feel authorities should restrict travel to and from COVID-19 areas (50.0%). Despite the above average positive attitude held by the participants, there were indications that persons with special needs in Nigeria were dissatisfied with the way the government treat them, especially during the COVID-19 outbreak and lockdown.

## Implications for counselling and social policy

Participants’ responses to questions 30 and 31 are informative and have implications for counselling and social policy by the government. Item 30 states “I feel it is hard getting palliatives/financial and material support/help from others during COVID-19 lockdown”, while item 31 states “I feel frustrated by the uncaring attitudes of the government towards people living with disabilities during COVID-19 lockdown”. These items were rated 3.31 and 3.28, meaning that most participants were in agreement with the questions and were dissatisfied with the manner in which society and especially the government has treated them during the COVID-19 outbreak and lockdown in the country. They acknowledged that they feel “frustrated and discouraged” about societal attitudes towards those with special needs. It therefore behooves the government to do the needful by adopting the WHO standard guidelines for persons with disabilities during the COVID-19 outbreak. Inclusive health practices are advocated by WHO and should be adhered to achieve fairness and equity. Social policy implication could focus around the need for the government to prioritize provision of social, economic and emotional support for persons with special needs in Nigeria. There is need to reduce unemployment rate among persons with special needs therefore, vocational training for semi-skilled manpower has to be supported by the government. All these would assist to alleviate the burden of poverty, which is closely associated with most people with disabilities.

## Limitations to the study

Some eligible contacted participants were unable to participate in the study as a result of non-possession of Android phones, voice to text software in their computer devices or other assistive technologies that can facilitate the completion of the Google survey forms. These barriers affected the number of participants.

## Conclusion

The study has established that persons with special needs in Nigeria possess a high level of knowledge about COVID-19 general characteristics, symptoms, and prevention and control, with majority of the participants being Nigerians living with a hearing impairment or visual impairment. In addition, persons with special needs have moderately positive attitude towards the COVID-19 pandemic but expressed dissatisfaction with the unfair treatment of the government towards them during the COVID-19 pandemic.

## Recommendations

Based on the findings of the study the following recommendations are made:

- Special educators should collaborate with counselling psychologists to create widespread awareness on strategies for prevention of COVID-19 among persons with special needs. Counselling and social policy efforts should be targeted at addressing strategies for the preservation of hearing sensitivity, reduction of visual impairment and other disabilities. The government and special educators should also sensitize the public on the effects of noise pollution, violence in society, drug abuse and exposure to poor lifestyles that make individuals vulnerable to hearing impairment and other kinds of special needs conditions. Counselling should also tailor towards encouraging and enabling persons with special needs to form goal-oriented groups or associations for the purposes of advocacy for their own needs. When persons with special needs belong to different advocacy associations or groups, the government can reach them through these organizations for more organized access to public assistance, services and palliatives. Furthermore, special educators and counselling psychologist should be co-opted as members of various COVID-19 task force committees established at both state and national levels. This will create an avenue for them to enlighten the government on how to provide meaningful welfare packages including befitting education to persons with special needs during this period of infection spread in the county.
- Provision of personal protective items such as nose masks, detergents for hand washing and cleaning of surfaces, gloves, and prescription drugs to boost personal immunity, maintain healthy daily living, independence, self-sustenance among persons with special needs during COVID-19 pandemic should be prioritized by the government. In addition, the government should make appropriate provisions for persons with special needs with respect to how they will access palliatives during the COVID-19 outbreak and lockdown. The reason is obvious, these individuals are vulnerable members of society; they find it difficult to perform certain functions that other individuals do with ease, such as communication, movement, cognitive activities to mention a few. Sometimes they rely on assistance from other people around them. A situation where government has no special provisions for individuals with special needs could expose them to further vulnerability. The government should desist from encouraging the culture of excluding the excluded.
- Government should intentionally create avenues for gainful employment of eligible and qualified persons with special needs into appropriate positions in society without discrimination. Vocational training among interested persons with special needs should be encouraged by the government. This will help in reducing the negative economic impact of COVID-19 such as substandard living conditions and poverty among some persons with special needs. Similarly, the Nigerian government as well as philanthropists should make funds available for the purchase of modern assistive technology devices for persons with special needs. This will ease access to digital information on the COVID-19 pandemic.

## Data availability

### Underlying data

Figshare: Demographic factors, Attitude and knowledge of persons with special needs towards COVID-19 in Nigeria: Implications for counseling and social policy. https://doi.org/10.6084/m9.figshare.12589025.v2 ( Lazarus & Oluwole, 2020).

Data are available under the terms of the Creative Commons Attribution 4.0 International license (CC-BY 4.0).
