# Supplementary material for: Demographic factors, attitude and knowledge of persons with special needs towards COVID-19 in Nigeria: Implications for counselling and social policy
Source: Emerald Open Res. 2020 Sep 30;2:55. doi: 10.35241/emeraldopenres.13800.2 (PMC7537591; doi:10.35241/emeraldopenres.13800.2)
Supplement: Peer review history

# Peer review: Reviewer response for version 2

**DOI:** 10.21956/emeraldopenres.15030.r27153

**Published:** 2020-10-02

**Authors:** Olufemi Adigun

**Affiliations:** 1 University of Zululand, KwaZulu-Natal, South Africa

Once again, I appreciate the call to review the manuscript. I am glad to see that the authors have painstakingly reworked the background to the study and other comments earlier raised. On this note, I strongly recommend that the article ‘Demographic factors, attitude, and knowledge of persons with special needs towards COVID-19 in Nigeria: Implications for counselling and social policy’ can be indexed in the current form.

Is the work clearly and accurately presented and does it cite the current literature?

Yes

If applicable, is the statistical analysis and its interpretation appropriate?

Yes

Are all the source data underlying the results available to ensure full reproducibility?

Yes

Is the study design appropriate and is the work technically sound?

Yes

Are the conclusions drawn adequately supported by the results?

Partly

Are sufficient details of methods and analysis provided to allow replication by others?

Yes

Reviewer Expertise:

NA

I confirm that I have read this submission and believe that I have an appropriate level of expertise to confirm that it is of an acceptable scientific standard.

# Peer review: Reviewer response for version 1

**DOI:** 10.21956/emeraldopenres.14878.r27054

**Published:** 2020-09-09

**Authors:** Christiana Olubukola Dada

**Affiliations:** 1 Kwara State University, Malete, Nigeria

My sincere gratitude goes to the Editor for the opportunity given to serve as a Reviewer.

 The paper is timely I would like to applaud the authors for their innovation and contribution to knowledge in the field of Special Education. It is a novel effort.

 Abstract: The last sentence of the abstract should be rework to indicate the counseling and social implications stated in the body of the work clearly. As it is written, recommendation has been stated not the counseling and social implications.

 Introduction: the introduction has been able to describe in details the views  of the Researchers. The use of tenses in Paragraph 2 line 13 should be corrected. The sentence should be written as “The most affected group are those between 31-40”. Paragraph 5 line 9 “Nigerians have….’’

 The researchers should also discuss briefly other types of disabilities as indicated in the findings. Physical disabilities, Deaf Blindness, Emotional disorder, Speech and language disorder and multiple disorder should be discussed briefly even though the focus is on visual and hearing impairment.

 Research Questions: The research questions were stated in measurable terms which is very good for a study in Education.

 Methods: The method used was adequately described by the Researchers.

 Ethical statement: Line 4, remove ‘In all’. 

 Participants: ‘Only special needs persons’ should be written as ‘Only persons with special needs’.

 Results: The presentation of results was succinct. ‘it is revealed’ should be written as ‘It was revealed’. This should be noted in this section since the researchers are relating a past event.

 Recommendations: I wish to suggest that the recommendations should be in prose form.

 I hereby wish to suggest that this Paper be Approved. The minor corrections noted should be taken into consideration by the Researchers.

Is the work clearly and accurately presented and does it cite the current literature?

Yes

If applicable, is the statistical analysis and its interpretation appropriate?

Yes

Are all the source data underlying the results available to ensure full reproducibility?

Yes

Is the study design appropriate and is the work technically sound?

Yes

Are the conclusions drawn adequately supported by the results?

Yes

Are sufficient details of methods and analysis provided to allow replication by others?

Yes

Reviewer Expertise:

NA

I confirm that I have read this submission and believe that I have an appropriate level of expertise to confirm that it is of an acceptable scientific standard.

# Peer review: Reviewer response for version 1

**DOI:** 10.21956/emeraldopenres.14878.r27057

**Published:** 2020-09-01

**Authors:** Olufemi Adigun

**Affiliations:** 1 University of Zululand, KwaZulu-Natal, South Africa

Thank you for the opportunity given to me to review this manuscript which investigated demographic factors, attitude and knowledge of persons with special needs towards COVID-19 in Nigeria. No doubt Nigerians living with special needs are faced with a mirage of challenges. They are mostly excluded from many issues that of national importance and their voices is not always recognized. Over the years, persons with special needs especially these with hearing and visual impairment suffer greatly in health related issues. Unfortunately, COVID-19 has not spared them from all ugly incidences that is associated with the pandemic. It is on this note that the research efforts made by the authors is highly commended as it has brought to fore, the nature of COVID-19 in Nigeria as it relates to persons with special needs and government.

 While the study has brought forward some contributions to knowledge, it is worthy to note that some corrections need to be made in order to make the manuscript better. One major of such is that the introductory part was well crafted but it presented more of general knowledge of COVID-19 with a very scanty information on the construct of the authors’ independent variables (knowledge and attitude). It is recommended that the introductory section of the manuscript be looked into and be recrafted to accommodate extensive review of disabilities/special needs person vis-à-vis communicable disease or other literatures that had previously interrogated health plus disabilities.

 A few corrections/suggestions have been identified on the research questions.

 The methodology section is well crafted. 

 Results is well presented.

 Discussion of findings may be beefed up.

 Implication for counselling and social policy needs to be further interrogated as it seems to be the core of the authors argument.

 An annotated PDF of the article with more comments can be found here.

Is the work clearly and accurately presented and does it cite the current literature?

Yes

If applicable, is the statistical analysis and its interpretation appropriate?

Yes

Are all the source data underlying the results available to ensure full reproducibility?

Yes

Is the study design appropriate and is the work technically sound?

Yes

Are the conclusions drawn adequately supported by the results?

Partly

Are sufficient details of methods and analysis provided to allow replication by others?

Yes

Reviewer Expertise:

NA

I confirm that I have read this submission and believe that I have an appropriate level of expertise to confirm that it is of an acceptable scientific standard, however I have significant reservations, as outlined above.
